# Optimizing Denture Stability: A Digital Workflow for Managing Bimaxillary Flabby Ridges in a Complete Denture Fabrication

**DOI:** 10.1002/ccr3.71855

**Published:** 2026-01-19

**Authors:** Edgar Garcia, Carlos Alberto Jurado, Franciele Floriani

**Affiliations:** ^1^ Department of Prosthodontics University of IOWA Iowa City Iowa USA; ^2^ Division of Operative Dentistry, Department of General Dentistry The University of Tennessee Health Science Center College of Dentistry Memphis Tennessee USA; ^3^ School of Dental Medicine Ponce Health Sciences University Ponce Puerto Rico USA

**Keywords:** complete dentures, computer‐aided design, denture design, geriatric patients

## Abstract

An integrated impression technique combining mucocompressive and functional approaches, supported by a digital workflow and closed‐mouth 3D‐printed prototypes, can effectively manage flabby ridges and mobile soft tissues. This method enhances denture stability, improves treatment efficiency, and increases patient satisfaction in complete denture fabrication.

## 1 Introduction

Retention, stability, and support are fundamental elements crucial to the success of complete dentures [1]. Clinical circumstances can significantly challenge the rehabilitation process, particularly in the lower jaw, which often poses a difficult scenario for clinicians seeking predictable outcomes [2]. This complexity arises from factors such as reduced residual ridge height, dynamic tongue mobility, and the presence of soft tissues that undergo dimensional changes during mandibular movements [3]. While upper complete dentures generally show positive outcomes, challenges may arise in cases involving excessively mobile soft tissues, or “flabby tissues” [4], this condition can cause dentures to displace anteriorly during occlusion, leading to rotation of the denture base, misalignment of the post‐dam area, and a subsequent loss of both stability and retention [5, 6]. Although studies demonstrate a high prevalence of flabby ridge in the maxilla [6, 7], this condition can also occur in edentulous mandibles, further complicating treatment. While pre‐prosthetic surgery to remove the flabby tissue may be an option, for patients where surgery is not feasible, accurate impression techniques and a stable occlusal scheme become crucial for achieving optimal outcomes [8].

Traditional Impression techniques for complete dentures typically follow a two‐step approach, an initial impression for diagnosis and custom tray fabrication, followed by a final impression. The final impression techniques include several methods, such as mucostatic, neutral zone, functional, mucocompressive, and selective pressure approaches [9]. To address mobile tissues, selective pressure and mucostatic techniques are often performed using custom tray designs that capture the tissue in a static position, ensuring an accurate and balanced impression without undesired tissue compression [9, 10].

In 2012, Dr. Jiro Abe introduced an innovative method for both maxillary and mandibular arches impressions to manage the challenges posed by mobile soft tissues [10]. For the maxilla arch, Abe's technique describes a mucocompressive technique that displaces flabby tissue posteriorly, creating a functional impression that stabilizes tissue, minimizes movement during mastication, and enhances denture retention while reducing inflammation. For the mandible, his functional impression method achieves a peripheral seal even with mobile tissues, enabling predictable outcomes without the need for pre‐prosthetic surgery. These techniques have become widely adopted, significantly improving patient comfort and functionality [11].

Moreover, the adoption of digital workflows in complete denture fabrication has proven highly advantageous. By increasing precision and reducing dimensional inaccuracies inherent in traditional acrylic‐based methods, digital protocols significantly shorten treatment times and reduce the number of necessary appointments [12]. This evolution in technique not only enhances clinical outcomes but also increases patient satisfaction [7, 12].

The purpose of this case report is to describe the treatment of a patient presenting with flabby ridges, using a combined mucocompressive impression technique for the upper arch and a functional impression technique for the lower arch, integrated with digital tools.

## 2 Case History/Examination

**FIGURE 1:**
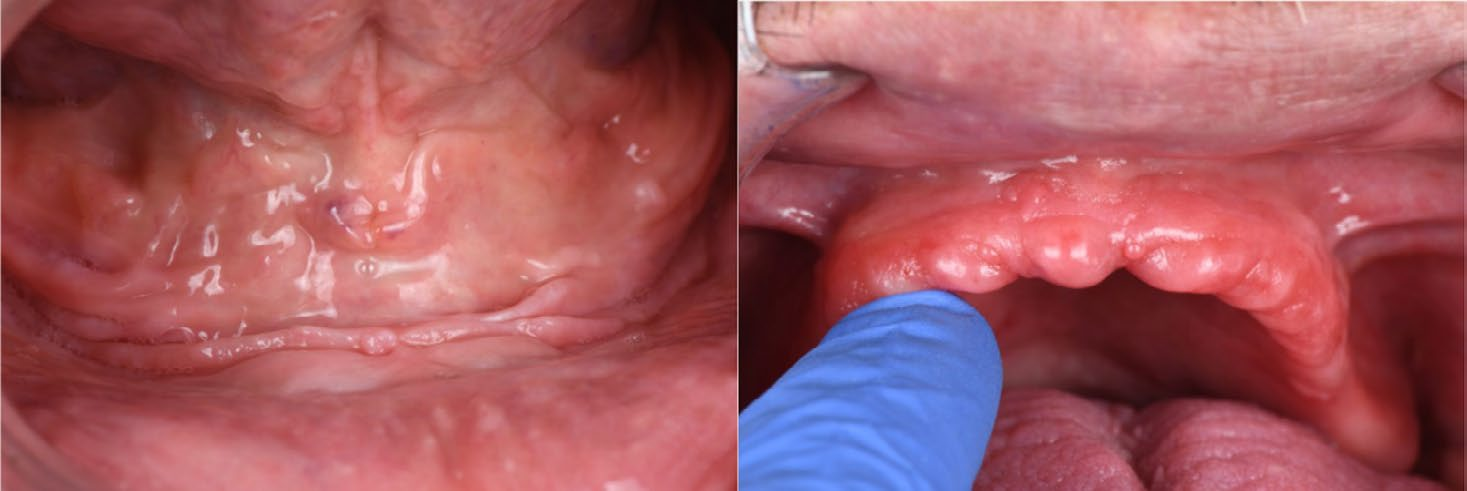
Intra‐oral images of upper and lower arch showing Flabby Ridges and ridge morphology.

**FIGURE 2:**
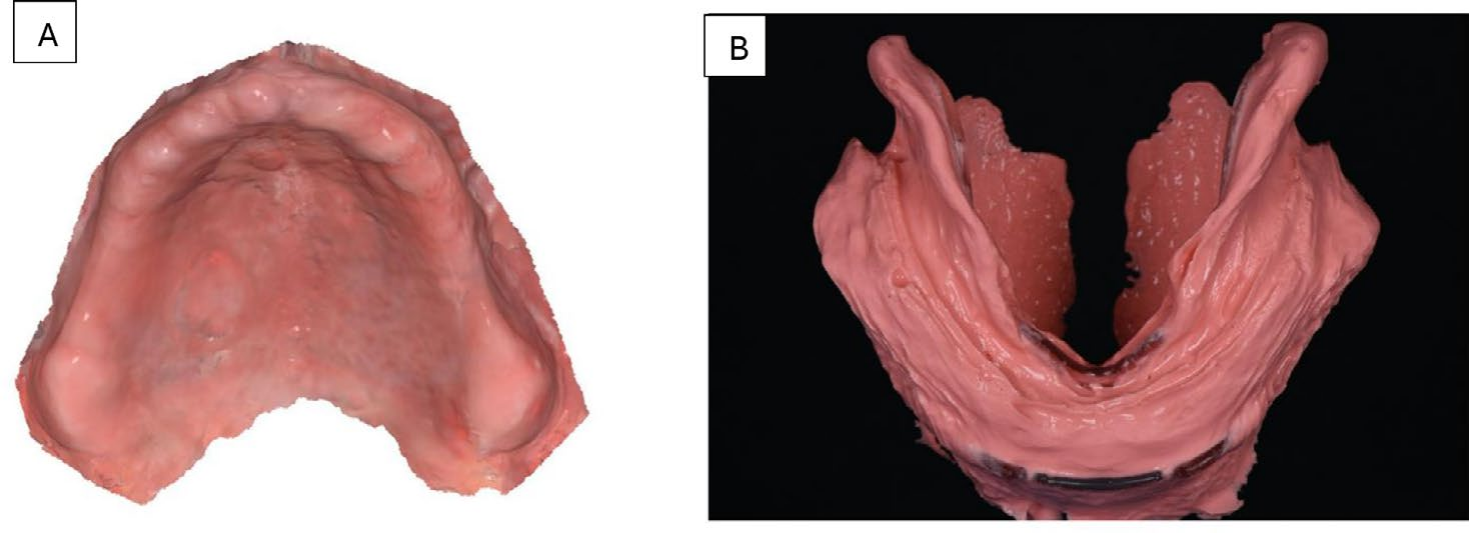
Preliminary intra‐oral digital impressions. (A) Maxillary intraoral digital impressions. (B) Mandibular digital impression with 3D‐printed frame cut back (FCB) tray technique.

A 66‐year‐old male presented to a private practice dental clinic with the chief complaint that his current complete dentures lacked retention and stability, making it difficult for him to eat. Patient's medical history was noncontributory, with no contraindications for dental treatment. An intraoral examination revealed a significant area of flabby tissue (Figure 1) in the upper maxillary arch, along with extensive bone resorption (Figure 2A). In the lower mandibular arch, a severely resorbed edentulous ridge was observed, featuring flabby tissue and a prominent spongy lingual area (Figure 2B). Excessive movement of both denture bases during functional activities highlighted the need for a more comprehensive treatment approach.

## 3 Differential Diagnosis, Investigations and Treatment

Patient was offered different options including implant overdentures, implant supported fixed prosthesis, and complete dentures; however, due to finances, he only required a new set of complete dentures. Patient was informed of the option to fabricate a try‐in in white color in order to evaluate it prior to the final prostheses, and he accepted.

**FIGURE 3:**
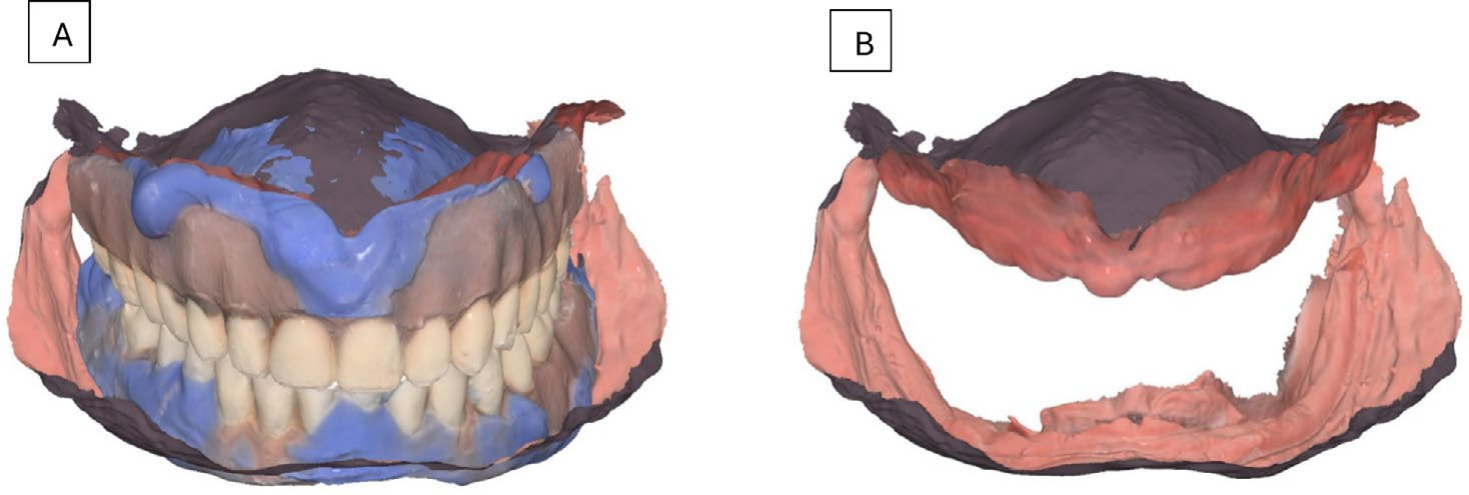
Primary occlusal registration. The relined dentures, maintaining the patient's occlusion at the established vertical dimension, were digitally scanned. (A) Preliminary intra‐oral scans aligned with relined previous dentures. (B) Maxillary and mandibular scans with the VDO of the previous dentures.

During the first appointment, the maxillary arch was scanned intraorally (Figure 3A), and a preliminary mandibular impression was obtained using a 3D printed Frame Cut Back (FCB) tray with irreversible hydrocolloid impression material (Cavex cream alginate, Cavex; Netherlands). The patient was guided to gently close his mouth until his lips met, ensuring the mandible remained in a resting position. To prevent excess impression material from accumulating in the buccal shelf area, the clinician carefully massaged the patient's cheeks and waited for the material to fully set (Figure 3B). Once complete, the impression was digitally scanned using an intraoral scanner (Medit i700, Medit, Korea).

**FIGURE 4:**
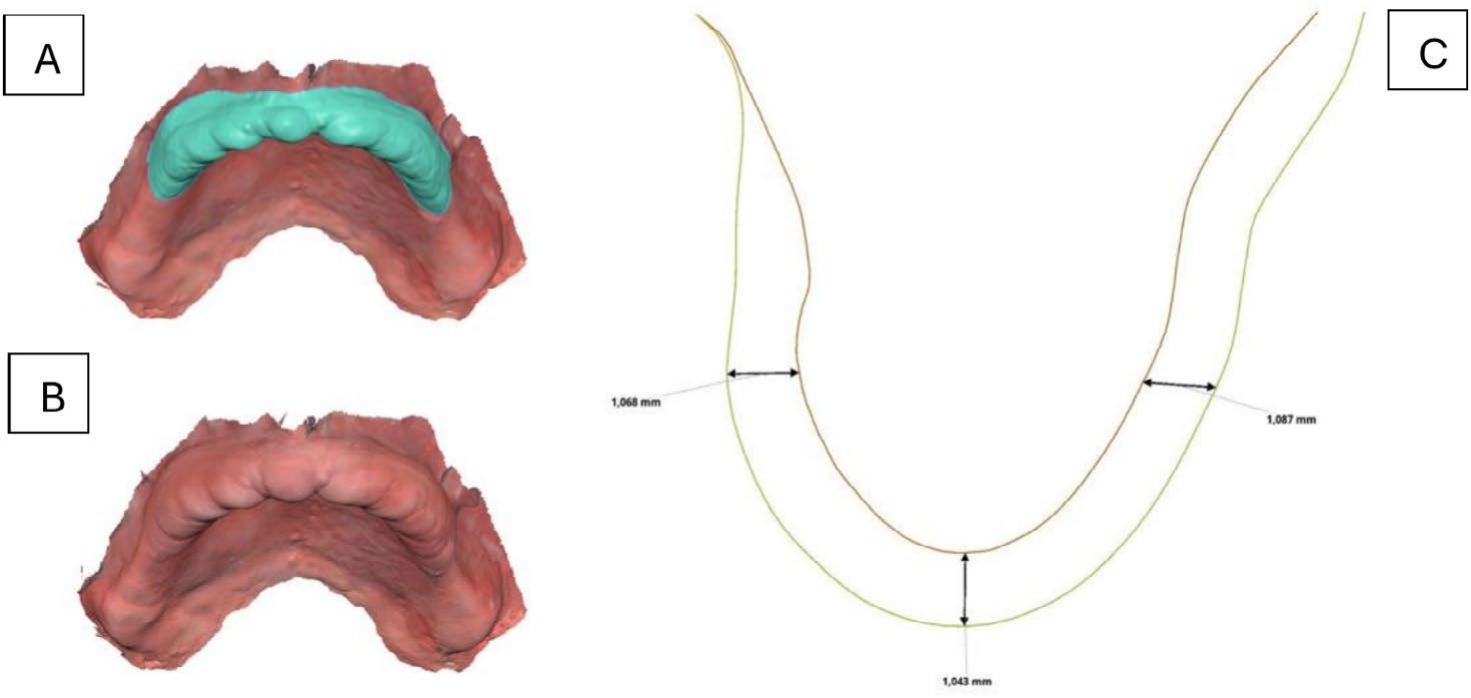
(A) Flabby ridge extension in the maxillary intra‐oral scan selected. (B) Flabby ridge area thickened in the design software. (C) Space created for the mobile tissue for making the custom tray/prototype for the wash impression.

**FIGURE 5:**
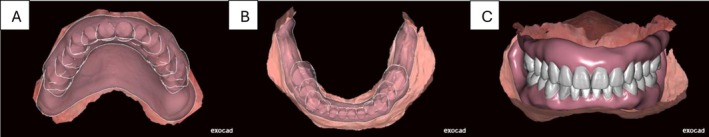
(A) Extension of the maxillary custom tray complete denture prototype. (B) Extension of the mandibular custom tray complete denture prototype. (C) Design of the custom trays' complete denture prototypes.

On the same appointment, a closed‐mouth relining was performed using light‐body impression material on the patient's existing complete dentures. This step obtained detailed anatomical data from the intaglio surfaces, guaranteeing accurate alignment with the scanned maxillary and mandibular arches throughout the digital workflow. Both the dentures and the patient's occlusion, maintained within the established vertical dimension, were scanned. In the design software (Medit Link, Medit, Seoul, Korea), the upper maxillary scan and the alginate mandibular scan were aligned with the denture scans (Figure 4). Once aligned, the flabby ridge area in the maxillary scan was digitally thickened to create space for impression material, enabling tissue displacement during border molding while keeping the tissue in a posterior position (Figure 5). The data were then imported into a separate design program (DentalCAD; Exocad GmbH, Germany), where prototypes were designed for closed‐mouth impression‐taking.

**FIGURE 6:**
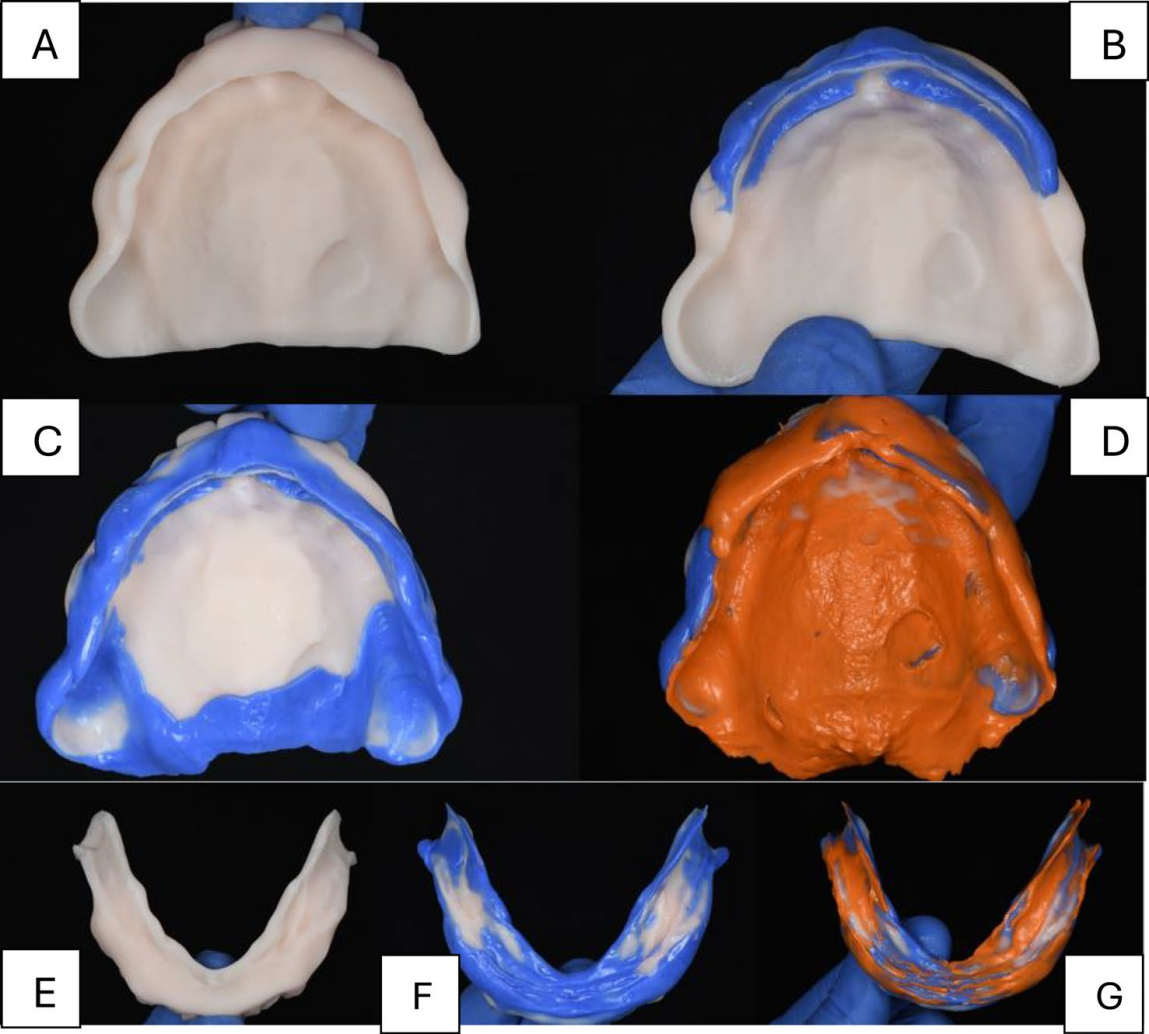
Step by step for the maxillary wash impression procedures. (A) Intaglio of the maxillary custom tray complete denture prototype. (B) Maxillary anterior border molding. (C) Maxillary posterior border molding. (D) Maxillary wash impression. (E) Intaglio of the mandibular custom tray complete denture prototype. (F) Mandibular border molding. (G) Mandibular wash impression.

To ensure the accurate registration of the border molding and final impression, the maxillary 3D‐printed prototypes customized tray was designed following specific parameters: first determine the outline in the posterior border and hamular notch areas. Include the maxillary tuberosity and avoid sensitive areas like the buccal frenum. The outline should be marked about 2 mm above the mucobuccal fold to account for frenum movement. In the anterior (labial) area, the outline should be made at the same level where the flabby ridge begins. Finally, extend the outline slightly posterior to the posterior palatal seal, “ah” line for proper tray extension with digital teeth arrangements (Figure 6A). For the mandibular 3D‐printed tray design, the outline extended fully to cover the retromolar pad, avoided Someya's sinew string, reached the deepest point of the buccal shelf, relieved pressure on the buccal frenum, and extended two millimeters beyond the mylohyoid ridge (Figure 6B). This design provided the necessary space for precise border molding and impression‐taking, ensuring optimal clinical results. The prototypes were then 3D‐printed using a biocompatible resin (KeyDenture Try‐In; Keystone Industries, USA) and verified intra‐orally to make sure there were no overextensions.

**FIGURE 7:**
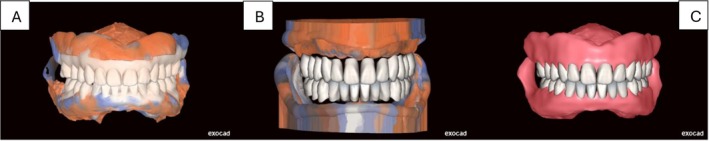
Complete dentures digital designs. (A) Intra‐oral scans of the wash impressions. (B) Tooth arrangement in the design software to maintain the vertical occlusion dimensions. (C) Complete dentures final digital design.

**FIGURE 8:**
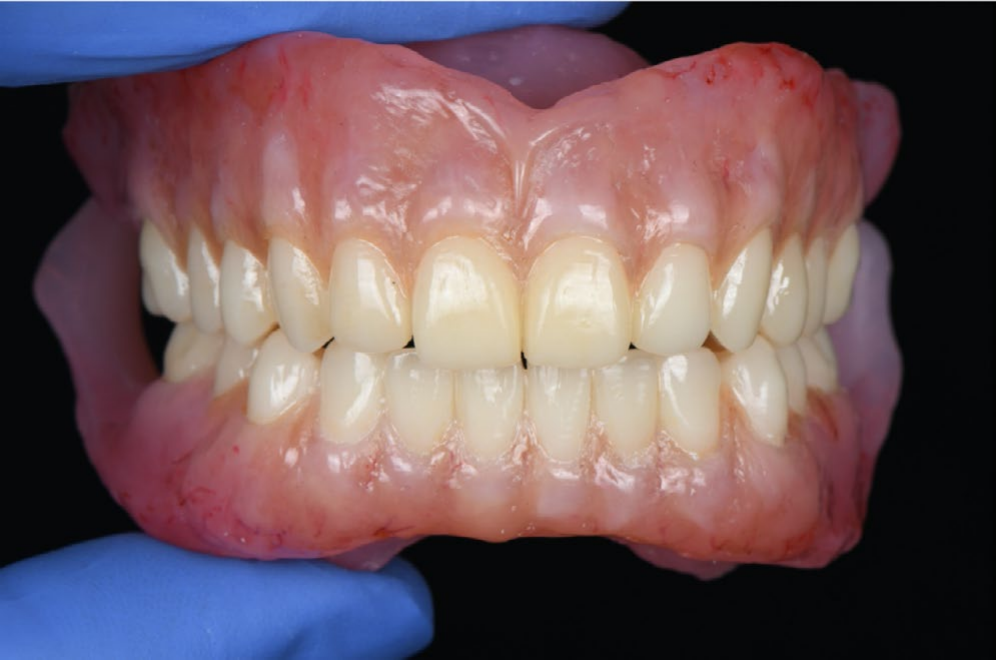
3D‐printed complete dentures after esthetic characterization.

During the final impression appointment, tray adhesive (Adhesive Polysiloxane; Coltène Holding AG; Switzerland) was applied to the 3D‐printed prototypes customized tray and allowed to dry. For the maxillary arch, heavy‐body polyvinyl siloxane (VPS) impression material (Heavy Body VPS; PlastCare USA) was applied to the anterior section of the tray, extending across the entire flabby ridge area. The tray was then carefully positioned in the mouth with posterior pressure, and the patient was asked to gently occlude to stabilize the tray and ensure proper tissue displacement. The remaining posterior border molding was completed in a single step using the same heavy‐body material by gently pulling the buccal mucosa downward in the posterior region and instructing the patient to move his mandible side to side to avoid interference with the condyloid process. Finally, a wash impression was taken using light‐body VPS (Light Body VPS; PlastCare USA) with a closed‐mouth technique (Figure 7). For the mandibular arch, border molding of the 3D‐printed prototypes customized tray was performed with heavy‐body vinyl polysiloxane (VPS) impression material (Heavy Body VPS Plastcare; USA). The patient was instructed to move his tongue from side to side to capture tongue movements accurately, followed by swallowing to record the mentalis muscle in its active state. Finally, with his mouth closed, he was directed to push his tongue towards the incisive papilla three times to imprint the floor of the mouth under tension, reflecting the contracted state of the mylohyoid muscle. A wash impression was obtained using light‐body vinyl polysiloxane (Light Body VPS, Plastcare; USA), with the patient repeating the same movements as during the border molding steps (Figure 8). The final impressions were digitally scanned, and the complete denture bases were designed using DentalCAD software (DentalCAD 3.2 Elefsina; exocad GmbH). The patient was informed the prototype was fabricated as a try‐in in white color in order to evaluate it prior to the final prostheses, and he accepted the teeth design.

**FIGURE 9:**
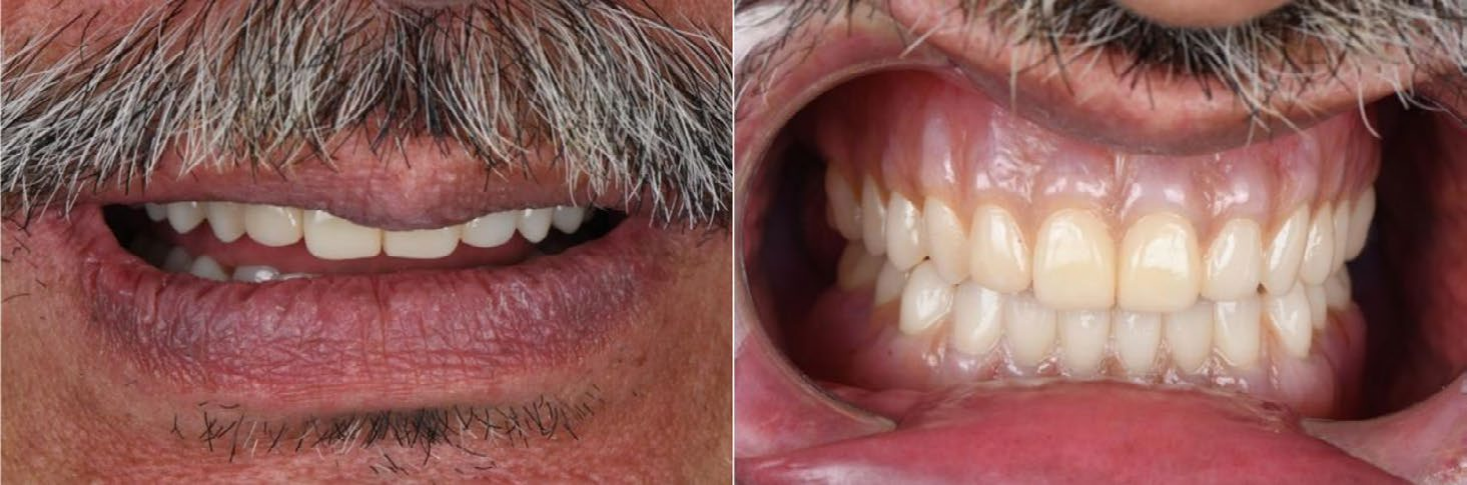
Extra‐oral images of 3D‐printed complete dentures.

The dentures were 3D‐printed with pink base resin (Denture Base Resin, Formlabs), while the teeth were fabricated using denture teeth resin in shade A2 (Premium Teeth Resin, Formlabs). Both the base and teeth were further characterized with multiple shades of light‐curing composite (SR Nexco, Ivoclar) to enhance esthetics. The retention, midline, esthetics, phonetics, lip support, buccal corridors, and occlusion were evaluated, and the patient was satisfied (Figure 9). The patient was instructed to return for follow‐up appointments to ensure continued comfort and function.

## 4 Discussion

In cases involving mobile tissues in the maxilla, impression‐making poses significant challenges, leading to the development of various management techniques [3, 7]. One widely used approach is a custom tray with an open window combined with selective pressure impression techniques to minimize displacement of flabby tissue [13, 14]. Song Yi Park proposed a hybrid technique that integrates an intraoral scanner with conventional impression methods. This approach uses the scanner's ability to capture the tissue in a static, uncompressed state, creating a digital model that preserves the natural contour without distortion. Despite this innovation, most techniques for managing mobile tissues focus on preventing anterior displacement during the impression process [8, 15].

Hypertrophic tissue in the maxilla is prone to forward displacement due to occlusal forces from opposing teeth. To address this, Jiro Abe et al., introduced a mucocompressive technique that differs from previous methods [16]. This approach involves fabricating a custom tray based on a primary alginate impression, with wax relief applied over the flabby tissue area. Finger pressure is applied to displace the tissue posteriorly during the anterior border molding, followed by the border molding of the posterior area. Small relief holes in the tray's flabby ridge region allow impression material to flow through, preventing excessive compression. The process is concluded with a closed‐mouth final impression. This method improves prosthesis retention and stability and, when paired with a stable occlusion featuring strong posterior contacts and a 2 mm overbite, promotes tissue stabilization and healing, gradually reducing tissue size [16].

Managing the edentulous mandible requires a distinct approach. Historically, conventional complete dentures in the mandible have been expected to fall short in fully restoring function and quality of life [17, 18]. Dr. Jiro Abe introduced a technique that diverges from conventional methods for fabricating complete dentures. In his technique, he begins with an irreversible hydrocolloid material and an FCB‐type tray for primary impressions [19], featuring open spaces at crucial support zones of the mandible, such as the buccal shelf and retromolar pad. Final impressions are made with a custom tray, using heavy‐consistency vinyl polysiloxane (VPS) for peripheral sealing and a light‐body silicone for the final wash impression, both applied with a closed‐mouth technique. Abe's technique aims to functionally replicate how mobile soft tissues adapt to edentulous surfaces, ensuring an effective seal [19].

The present clinical report outlines a modified impression technique for both arches based on Abe's methods. The author believes that the key lies in the primary registrations of both arches. For the maxillary arch, scanning the mobile tissue in a static state, rather than using alginate as in the original technique, allows for more controlled posterior compression. This reduces the need for excessive backward pressure, as the custom tray's slight space and the impression material consistency provide an appropriate amount of posterior pressure, stabilizing the tissue and preventing anterior displacement during the final impression. For the mandibular arch, taking the impression with alginate injected while the patient closes their mouth captures not only the peripheral tissues but also those over the ridge in a functional state. Scanning the alginate immediately, without proceeding to the casting process, provides greater accuracy in reproduction compared to traditional casting, which may introduce slight dimensional changes. This combination of traditional methodologies with digital technology provides a personalized and effective dental solution for this patient with mobile tissue.

Moreover, the dentures were 3D printed, resulting in fewer appointments and greater adaptation, precision, enhancing predictability compared to conventional denture processing techniques [20, 21, 22]. These findings have substantial clinical implications. For practitioners, adopting digital technology for denture fabrication could mean more predictable and satisfactory outcomes for patients. Additionally, the ability to maintain occlusion and provide better force distribution could decrease the need for frequent adjustments, thus reducing overall treatment time and improving patient satisfaction. This study's exploration of digital and conventional denture fabrication techniques highlights the potential for improved efficiency and patient outcomes with the integration of new technologies. Despite these promising results, the dental practitioners continue to explore and validate the long‐term effectiveness and reliability of 3D‐printed and milled dentures. Future research should focus on the durability of materials used in 3D printing, the long‐term stability of the dentures, and patient‐based outcomes across diverse demographics to solidify the position of 3D printing as a superior method for denture fabrication.

## 5 Conclusion

An edentulous patient with a bimaxillary flabby ridge was managed using a combined mucocompressive‐functional impression technique for the maxillary arch and a functional impression technique for the mandibular arch. The dentures were digitally designed and 3D printed, enhancing precision and predictability. Integrating these impression techniques with digital workflows allowed for the fabrication of dentures with excellent adaptation, retention, and stability. At follow‐up appointments, the patient reported high satisfaction with both esthetics and function.

## Author Contributions

**Edgar Garcia:** conceptualization, data curation, resources, visualization, writing – original draft, writing – review and editing. **Carlos Alberto Jurado:** methodology, writing – original draft, writing – review and editing. **Franciele Floriani:** supervision, writing – original draft, writing – review and editing.

## Funding

The authors have nothing to report.

## Ethics Statement

According to the institutional and national research ethics guidelines, formal ethical approval was not required for this single‐patient case report. Written informed consent was obtained from the patient for treatment and publication of anonymized clinical information and images.

## Data Availability

The authors have nothing to report.
